# Multilevel Spatial Structure Impacts on the Pollination Services of *Comarum palustre* (Rosaceae)

**DOI:** 10.1371/journal.pone.0099295

**Published:** 2014-06-10

**Authors:** Laurent Somme, Carolin Mayer, Anne-Laure Jacquemart

**Affiliations:** Earth and Life Institute-Agronomy, Research Team Genetics, Reproduction, Populations, Université Catholique de Louvain, Louvain-la-Neuve, Belgium; University of Arizona, United States of America

## Abstract

Habitat destruction and fragmentation accelerate pollinator decline, consequently disrupting ecosystem processes such as pollination. To date, the impacts of multilevel spatial structure on pollination services have rarely been addressed. We focused on the effects of population spatial structure on the pollination services of *Comarum palustre* at three levels (i.e. within-population, between-populations and landscape). For three years, we investigated 14 Belgian populations, which differed in their within-population flower density, population surface, closure (i.e. proportion of the population edge that consisted of woody elements) and isolation (i.e. percentage of woody area cover within a 500 m radius from the population centre). We tested whether these spatial characteristics impact on pollinator abundance and visitation rate and thus, reproductive success of *C. palustre*. Insects were observed in 15 randomly-chosen plots in each population. We tested for pollen limitation with supplemental hand-cross pollination. Bumble bees and solitary bees were the major pollinators through all populations. Within populations, plots with high flower densities attracted high numbers of bumble bees and other insects. High bumble bee and solitary bee abundance was observed in populations presenting high proportions of woody edges and in populations within landscapes presenting high proportions of woody areas. Seed set resulting from open pollination varied with bumble bee and solitary bee visitation rate, leading to increased pollen limitation when pollinators were scarce. Since the reproductive success depended on the visitation rate of the main pollinators, which depended on multilevel spatial structure, wetland management plans should pay special attention to favour a mosaic of biotopes, including nesting sites and food resources for insects. This study particularly supports the relevance of a mix wetlands and woody habitats to bees.

## Introduction

A general decline in pollinators has been observed over the past few decades [1], [2]. Since a decrease in their abundance and diversity could entail the disruption of pollination services [3], their loss could lead to what has been described as a global pollination crisis [1], [4]. Animal-mediated pollination plays an important functional role in most terrestrial ecosystems and is vital to the maintenance of plant communities and the conservation of global diversity [1], [5], [6]. About 80% of wild plant species in temperate regions depends on insects, mainly bees, for pollination [7], [8]. Insect-pollinated plant species might suffer from reduced visits of pollinators resulting in pollen limitation [1], [9], [10]. As a consequence, reproductive success of plants with a pollination deficit would be limited since plants produce fewer fruits and/or seeds than they would with adequate pollen transfer [11]–[13]. Scarcity of mates for cross-pollination resulting from reduced abundance of flowering individuals might further increase pollen limitation [14]–[16]. Moreover, pollinators are usually attracted by large floral displays (e.g. high flower density), and patches of low flower density risk receiving fewer pollinator visits [17], [18]. Therefore, understanding the factors which influence pollination service is fundamental for the conservation of biodiversity [19].

Over the last century, the intensification of anthropogenic activities due to the rapid growth of the human population and the concomitant abandonment of traditional agropastoral activities led to large-scale land-use changes [20]. Natural and semi-natural habitats consequently suffered from severe destruction and fragmentation threatening biodiversity [21], [22]. Beside reduction of habitat area, fragmentation modifies and reduces connectivity and quality of remaining fragments by increasing isolation and edge effects [23], [24]. These changes in habitat properties might be accompanied by species' extinctions leading to simplification of both plant and animal communities [4], [25], [26]. The decrease in plant population surface and/or plant density might negatively affect pollinators as the quantity or quality of floral resources – pollen and/or nectar – is reduced [1], [27], [28]. Depending on their dispersal abilities, increased isolation of plant populations within a hostile matrix might further limit pollinator availability and disturb their foraging behaviour [29]. In particular, woody areas could act as potential barriers to pollinators [30], [31]. On the other hand, edges of woody areas might represent suitable nesting sites for solitary bees and bumble bees, for instance [32]–[34], since they exploit several biotopes to meet their food and nesting requirements [8], [30], [35]. Plant populations as potential foraging zones need to be within foraging range from such suitable nesting sites [36], [37]. The impact of woody areas on insects, solitary bees and bumble bees particularly, in fragmented open semi-natural biotopes has often been neglected in temperate regions (but see [8], [30], [32], [38], [39]). Since insects show variable habitat requirements (i.e. foraging, mating and nesting sites) and mobility depending on spatial structure, synergies occurring within multilevel spatial structure should get more attention when studying pollination services.

Wet meadows and peat bogs represent particularly threatened biotopes [40], [41], which suffer from severe destruction and fragmentation due to land use modification [41], [42]. In Belgium, their extent has decreased by 90% since the middle of the 19^th^ century [43], [44]. Nowadays, they occur as fragments bearing small native plant populations that are isolated in a mosaic of inhospitable biotopes [45]. Most studies investigating habitat loss and fragmentation in Europe focus on plant and insect species occurring in agricultural landscapes or forests, while species-rich open semi-natural biotopes such as wetlands are neglected [35], [40]. Considering the accelerating general decline of pollinators, there is a strong need to understand the role of multilevel spatial structure (i.e. within population, population and landscape levels) in affecting pollinator communities in order to develop efficient management and conservation strategies for pollinators and their plant partners in wetlands.

For three years, we studied abundance and visitation rates of insect visitors of *Comarum palustre* and plant reproductive success. *Comarum palustre* directly depends on insect pollination to set seed because spontaneous selfing is negligible [46]. The flowers are visited by a large array of insects, in particular Hymenoptera – mainly *Bombus* species – Diptera and Lepidoptera [47], [48].

Being an ecological process, pollination might depend on spatial scales much larger than a single biotope since insects respond to habitat fragmentation in contrasting ways [28], [39], [49]. Our aim was therefore to link spatial patterns and ecological processes at a multilevel spatial scale [49]. We specifically investigated if visitor abundance and visitation rates would be affected by changes in (1) within-population flower density, (2) population surface and closure (i.e. proportion of the population edge that consists of woody elements) at the population level and (3) population isolation (i.e. percentage of woody area cover within a 500 m radius of the population centre) at the landscape level. If so, (4) would such modifications in insect visitation rate lead to pollen limitation and reduced reproductive success?

## Materials and Methods

### Ethics statement

The “Département de la Nature et des Forêts” (DNF, Région Wallonne, Belgium) allowed us to sample plant and insect individuals in nature reserves, and the DNF and Natagora granted access to their properties.

### Plant species

*Comarum palustre* L. (Rosaceae) is a self-compatible, perennial herbaceous plant species, which occurs in pioneer stages of peat bogs, fens and wet meadows [47], [50]. The blooming period lasts from May to July [47]. The species reproduces sexually but also propagates clonally via long lignifying rhizomes [47], [51]. Plant individuals grow sympodially with a terminal inflorescence, on average bearing seven purple flowers. These bowl-shaped flowers last for about five days, where the female phase follows the male phase two days after anthesis [47]. The centre of the hypanthium is formed by a spongy receptacle that carries large numbers of single-ovuled carpels (227±71, mean ±SD, n = 113). A flower has about 20.0 (±0.2) anthers each producing about 19 000 pollen grains (range 13 761–23 453) [47]. Copious nectar is secreted between the base of the receptacle and the stamens. The daily production averages 9.5 µl (±5.8 µl) per flower with a mean sugar concentration of 31.5% (±9.3%) (52.0±1.5% fructose, 47.2±1.7% glucose, 0.9±0.3% sucrose, n = 7).

### Population spatial characteristics

**Figure 1:**
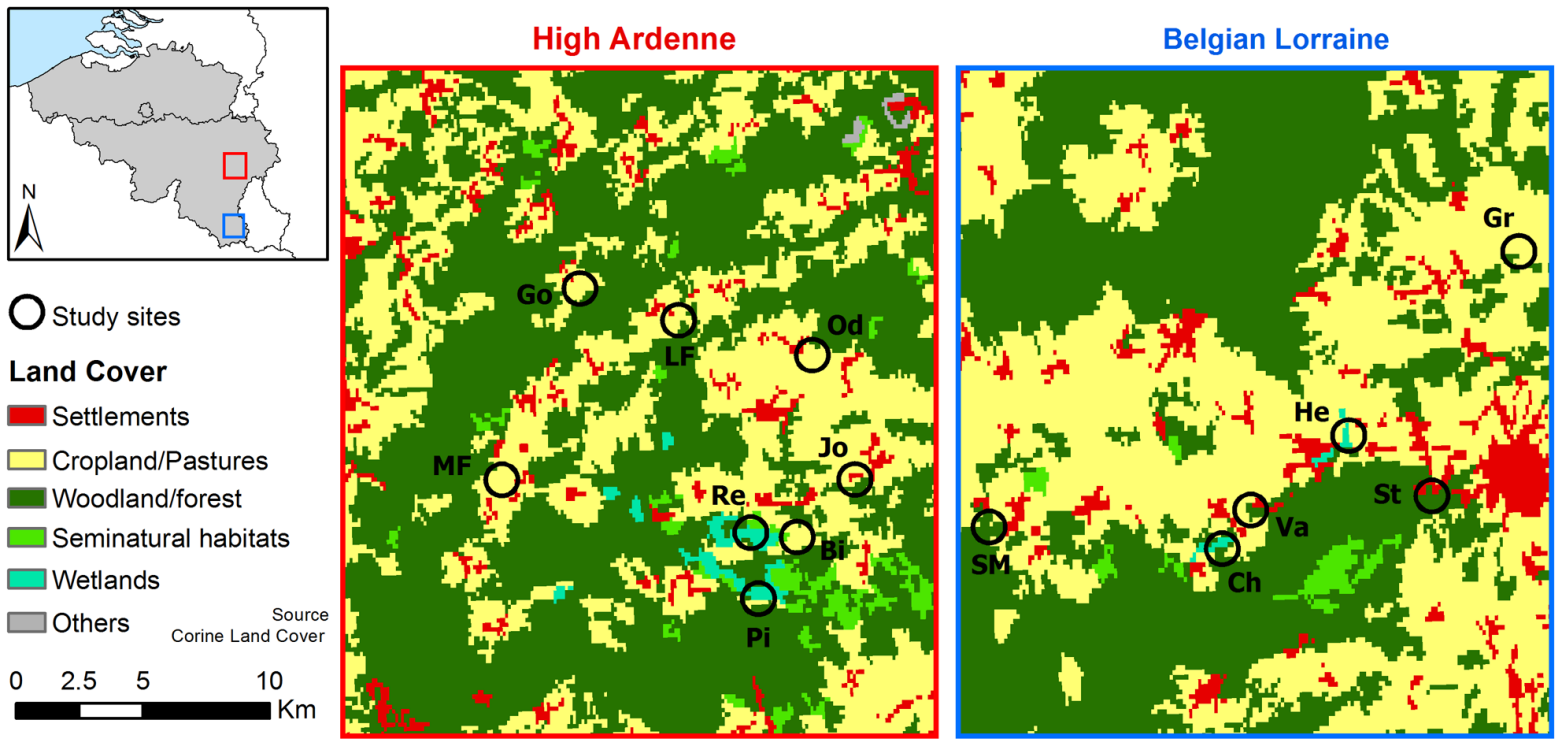
Study regions and study sites in High Ardenne (red square) and Belgian Lorraine (blue square), Belgium.

We studied pollinators and pollen limitation in 14 populations of *Comarum palustre* in High Ardenne and Belgian Lorraine (Figure 1). Populations were defined as all flowering individuals of *C. palustre* occurring in a wetland fragment. These populations were 1.0 up to 60.8 km apart and located at altitudes between 342 and 611 m above sea level (Figure 1). The study regions were characterized by semi-natural grasslands, fens, bogs and woody areas intermingled with urbanized and agricultural areas. Populations were mapped with the help of a GPS (Magellan sportrak pro). We refer to ‘population surface’ as the area covered by individuals of *C. palustre* within the wetland fragment (Table 1). Woody areas (e.g. willow, *Salix* spp., thickets, deciduous and mixed forests (mainly *Fagus sylvatica* and/or *Quercus robur* stands), and spruce (*Picea abies*) plantations) might represent potential barriers to pollinators, thus negatively influencing landscape connectivity [38], [52]. We therefore defined one factor ‘population isolation’ as the percentage of woody area cover within a 500 m radius from the centre of the population using ge-path v 1.4.6 (Cocoa Research Center, Bahia, Brasil). We preferred this area-based isolation metric to a distance-based metric since it allows incorporation of landscape characteristics and identifies population isolation from a pollinator perspective [6], [53], [54]. The population edge effect was defined as ‘population closure’, which was calculated as the proportion of the population edge that is constituted of woody elements. Within-population flower density refers to the mean number of open flowers recorded on 15 randomly chosen 1-m^2^ plots per population.

**Table 1:** Characteristics of the 14 Belgian populations of *Comarum palustre*.

Population	Coordinates	Flower density (flowers/m^2^)	Population surface (m^2^)	Population closure (%)	Population isolation (%)	N_obs_	Bumble bees		Solitary bees		Other insects	
							Number of individuals	Visitation rate	Number of individuals	Visitation rate	Number of individuals	Visitation rate
Joubiéval (Jo)	50°15′37.69" 5°50′20.78"	22±12	2261	13.6	25.4	30	31	0.13	3	0.01	61	0.12
Grendel (Gr)	49°45′14.56" 5°48′59.14"	17±10	1623	22.9	14.1	14	9	0.15	1	0.01	56	0.35
Fosse (MF)	50°15′45.77" 5°38′37.44"	41±28	327	23.2	23.0	24	80	0.32	2	0.06	14	0.02
Heinsch (He)	49°41′43.01" 5°43′46.71"	25±10	1529	34.3	30.2	16	1	0.002	8	0.02	137	0.40
Bra (LF)	50°19′5.51" 5°44′35.54"	45±34	468	36.1	29.3	21	82	0.28	1	0.01	34	0.05
Regné (Re)	50°14′32.57" 5°46′51.49"	14±9	58 860	43.7	20.7	40	20	0.09	3	0.01	45	0.07
Odrimont (Od)	50°18′18.00" 5°48′59.86"	19±12	5195	44.1	15.9	34	12	0.05	1	0.003	32	0.05
Stockem (St)	49°40′30.62" 5°46′14.19"	18±13	1241	45.3	58.7	41	71	0.29	6	0.07	111	0.22
Bihain (Bi)	50°14′25.89" 5°48′22.60"	14±9	945	51.1	8.4	25	31	0.18	6	0.05	10	0.04
Vance (Va)	49°40′17.91" 5°40′47.51"	23±15	7450	52.5	28.0	44	80	0.33	22	0.11	195	0.20
Pisserotte (Pi)	50°13′8.32"" 5°47′3.74"	20±14	184	60.5	58.7	38	77	0.35	5	0.03	47	0.08
La Gotale (Go)	50°19′48.02" 5°41′18.84"	6±4	154	62.1	43.5	14	5	0.05	3	0.15	11	0.10
Sainte-Marie (SM)	49°40′3.31" 5°32′56.81"	16±13	253	88.2	67.1	15	60	0.71	45	0.26	63	0.32
Chantemelle (Ch)	49°39′33.51" 5°39′54.89"	23±15	6422	89.8	73.7	44	115	0.40	27	0.05	106	0.20

“Flower density” is the mean number of open flowers recorded on 1-m^2^ plots (±SE; n = 15) per population. “Population surface” is the area covered by individuals of *C. palustre*. “Population closure” is the proportion of the population edge that is constituted of woody elements. “Population isolation” is the percentage of woody area cover in a 500 m radius from the centre of the population. The number of 10-min insect observation periods performed per population (N_obs_), abundance (i.e. total number of individuals) and mean visitation rate (i.e. ratio between the number of visited flowers and open flowers within 1-m^2^ plot) per 10-min insect observation periods of bumble bees, solitary bees and other insects are also provided. Observations were conducted for three years in seven populations (Joubiéval, Regné, Odrimont, Stockem, Vance, Pisserotte and Chantemelle), two years in four populations (Fosse, Bra, Bihain, La Gotale) and one flowering season in three populations (Grendel, Heinsch and Sainte-Marie). Populations are arranged by increasing population closure.

### Insect observations

Observations were conducted from 2010 to 2012 in seven core populations (Joubiéval, Regné, Odrimont, Stockem, Vance, Pisserotte and Chantemelle). Another four populations (Fosse, Bra, Bihain, La Gotale) were investigated for two years and three other populations for one year (Grendel, Heinsch and Sainte-Marie; Table 1). By doing so, we enlarged the range of spatial characteristics studied. All open flowers within 15 randomly chosen 1-m^2^ plots per population each year (established for flower density estimates), were observed for 10-min periods. We spread insect observation periods over two sunny and warm days per population during the peak of flowering in June, which lasted about two weeks. Flowers were observed for a total observation time of 72 h and 10 min. The minimum distance between two plots was about 3 m on one observation day and care was taken to spread the plots over the entire population. Insects were considered pollinators if they touched floral sexual organs. We identified pollinators in the field or captured them for further identification. Captured insect specimens were deposited in the insect collection of the Earth and Life Institute at the Université catholique de Louvain (Louvain-la-Neuve, Belgium). Due to their high morphological similarity, individuals of *B. terrestris*, *B. lucorum*, *B. cryptarum* and *B. magnus* were pooled into one operational taxonomic unit (OTU), *B. terrestris s.l.* [55]. We calculated flower visitation rate per 10-min as the ratio between the number of flowers visited and the number of open flowers per 1-m^2^ plot. We performed the analyses of abundance (i.e. number of individuals) and visitation rates for three distinct groups (i) bumble bees, (ii) solitary bees and (iii) other insects. We subdivided bees into two pollinator groups, bumble bees and solitary bees, since they differ greatly with respect to foraging distances and life history traits [36], [56], [57]. Since honey bees (2.1% of the visitors during observations) depended more on the activity of local bee-keepers than on habitat spatial structure, we excluded them from the analyses.

### Pollen limitation and reproductive success

Pollen limitation experiments were conducted in the same populations as insect observations from 2010 to 2012. For pollination treatments, we selected 10 plots of 1 m^2^ per population, which differed from insect observation plots. Each plot contained at least two inflorescences with the first flower of the cyme at female stage. By doing so, we avoided differences in seed set due to flower position within the inflorescence and within-ramet resource allocation [47], [58]. To test for pollen limitation, we performed supplemental hand-cross pollination with pollen from male-phase flowers situated at least 8 m away from the recipient, as results on genetic structure of *C. palustre* showed a genet size up to 7 m [59]. Pollen was carefully applied once by brushing the open anthers onto all the recipient stigmas. The first flower of the cyme of another inflorescence at the same stage was marked and left for open pollination within each plot. Both manipulated and unmanipulated flowers were marked by tying a piece of coloured wool, one colour per treatment, round the inflorescence.

Fruiting flowers were harvested three weeks later just before dehiscence to ensure the full development of the achenes, and stored in envelopes at room temperature before achenes were counted. Achenes were scanned at 600 dpi and counted using a macro created with ImageJ (National Institute of Mental Health, Bethesda, Maryland, USA). They were automatically sorted into three categories according to size and shape at the same time: plump achenes, partly unfilled achenes and unfertilized ovules. This morphological screening was ascertained by testing the achenes for viability [60]. They were longitudinally cut and soaked in a 1% 2,3,5-triphenil-tetrazolium chloride solution for 24 h at 35°C. The colorimetric test showed that 90% of the plump achenes were viable and 97% of the unfilled achenes were aborted. Results on seed set were adapted accordingly.

### Statistical analyses

No intercorrelations were detected between the different spatial characteristics (Spearman Rank Correlation, *r*_s_ = −0.18–0.28, P>0.05), except between population isolation and closure (Spearman Rank Correlation, *r*_s_ = 0.52, P<0.05). However, since woody areas influence insect abundance and visitation rate, and consequently plant reproductive success in opposite ways, we assessed woody area effects at both the landscape (i.e. population isolation) and population (i.e. population closure) levels. We defined spatial characteristics as influencing factors (fixed effects) and related the response variables (i.e. bumble bee, solitary bee and other insect abundance, visitation rate and seed set resulting from open and supplemental pollination) to them with generalised linear mixed models (GLMM). Observations were pooled within all populations and years. This leads to statistically non-independent data points (i.e. repeated observations) that can nevertheless be correctly analysed with GLMMs [61], [62]. Different observation years were nested within the populations and both included as random factors in the models [63]. For GLMMs with count data, we used a negative binomial error distribution, with a log link function and Laplace likelihood approximation to reduce overdispersion in the variance of the data. For proportional data, beta distribution with a logit function and residual penalized likelihood approximation resulted in the best model adaptation [64]. We log-transformed population surface to improve goodness of fit. “Type III Tests of Fixed Effects” tested the significance of each of the fixed effects specified in the model.

To assess the effects of pollination treatment on seed set (arcsin transformed), we performed a one-way ANOVA. If not indicated otherwise, data are presented as mean ± standard deviation. GLMMs were estimated with SAS 9.2 (“Proc GLIMMIX”; SAS Institute Inc., Cary, NC, USA); other analyses were computed with R 2.13.0 [65].

## Results

### Insect observations

A total of 1 771 insects from 63 different species were recorded visiting flowers of *C. palustre* (Table S1). The main visitors were Hymenoptera (49.1%), Diptera (35.7%) and Lepidoptera (9.2%). Other insect orders (Coleoptera, Mecoptera, Heteroptera, Hemiptera and Orthoptera) were recorded in negligible numbers. Hymenoptera were mainly constituted by bees, with 16 species representing 41.2% of all visitors. Bumble bees, with 10 different species, summed up to 96.1% of the bee visitors. Syrphidae (Diptera) accounted for 26.1% of all visitors with 9 different species.

Within-population flower density positively affected abundance of bumble bees and other insects, but not of solitary bees (Table 2). Population closure positively influenced bumble bee and solitary bee abundance since populations of *C. palustre* showing high proportions of woody edges contained high numbers of bumble bees and solitary bees. Isolated populations of *C. palustre*, i.e. located in landscapes with high proportions of woody areas, contained high abundance of bumble bees and solitary bees, as well. Population surface, closure and isolation had no significant influence on the number of other insects (Table 2).

**Table 2:** Effects of spatial characteristics on pollinator abundance and visitation rates.

	Bumble bees				Solitary bees				Other insects			
	Number of individuals		Visitation rate		Number of individuals		Visitation rate		Number of individuals		Visitation rate	
	d.f.	F	d.f.	F	d.f.	F	d.f.	F	d.f.	F	d.f.	F
Flower density	1, 399	16.67***	1,399	0.05	1, 399	0.27	1,399	0.83	1, 399	6.53*	1,399	3.29
Population surface	1, 399	0.21	1,399	0.20	1, 399	0.14	1,399	0.30	1, 399	0.31	1,399	0.18
Population closure	1, 399	39.11***	1,399	1.76	1, 399	11.45***	1,399	10.35**	1, 399	0.11	1,399	0.26
Population isolation	1, 399	2.13	1,399	5.59*	1, 399	5.15*	1,399	5.91*	1, 399	1.57	1,399	1.31

Influence of within-population flower density and population surface, closure and isolation on the abundance (i.e. total number of individuals) and visitation rate of bumble bees, solitary bees and other insects. (Degrees of freedom (d.f.) and F-values (F) of GLMMs for the 14 *Comarum palustre* populations; * P<0.05, ** P<0.01, *** P<0.001).

**Figure 2:**
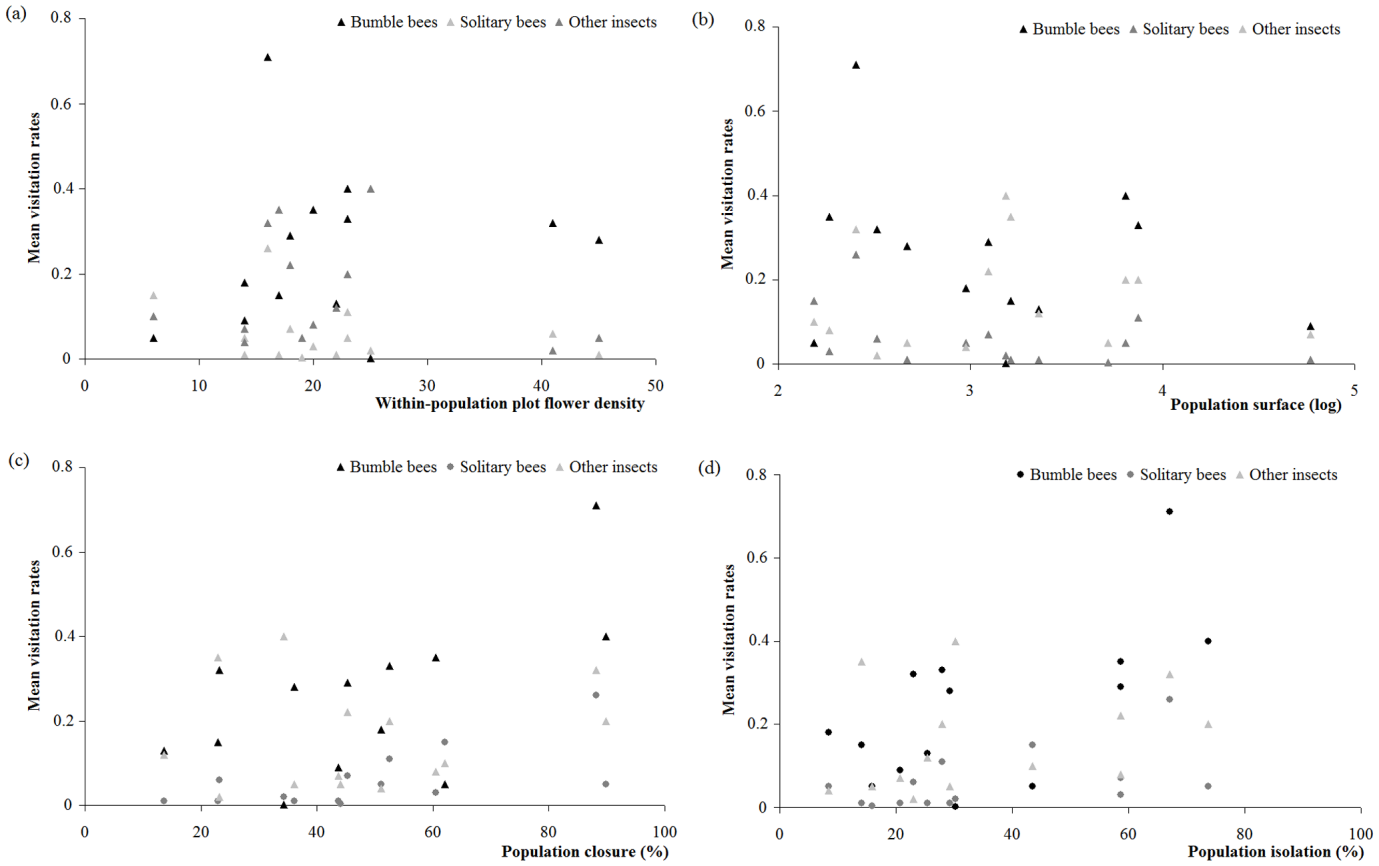
Effect of spatial characteristics on bumble bee, solitary bee and other insect visitation rate. Visitation rate of solitary bees, bumble bees and other insects according to (a) within-population plot flower density, (b) log-transformed population surface, (c) population closure and (d) population isolation. Significant and non-significant results are represented by filled circles and filled triangles, respectively.

No significant influence of within-population flower density on insect visitation rate was detected (Table 2; Figure 2). Population closure and isolation positively influenced solitary bee visitation rate, whereas only population isolation positively affected bumble bee visitation rate (Table 2; Figure 2).

### Reproductive success and pollen limitation

**Figure 3:**
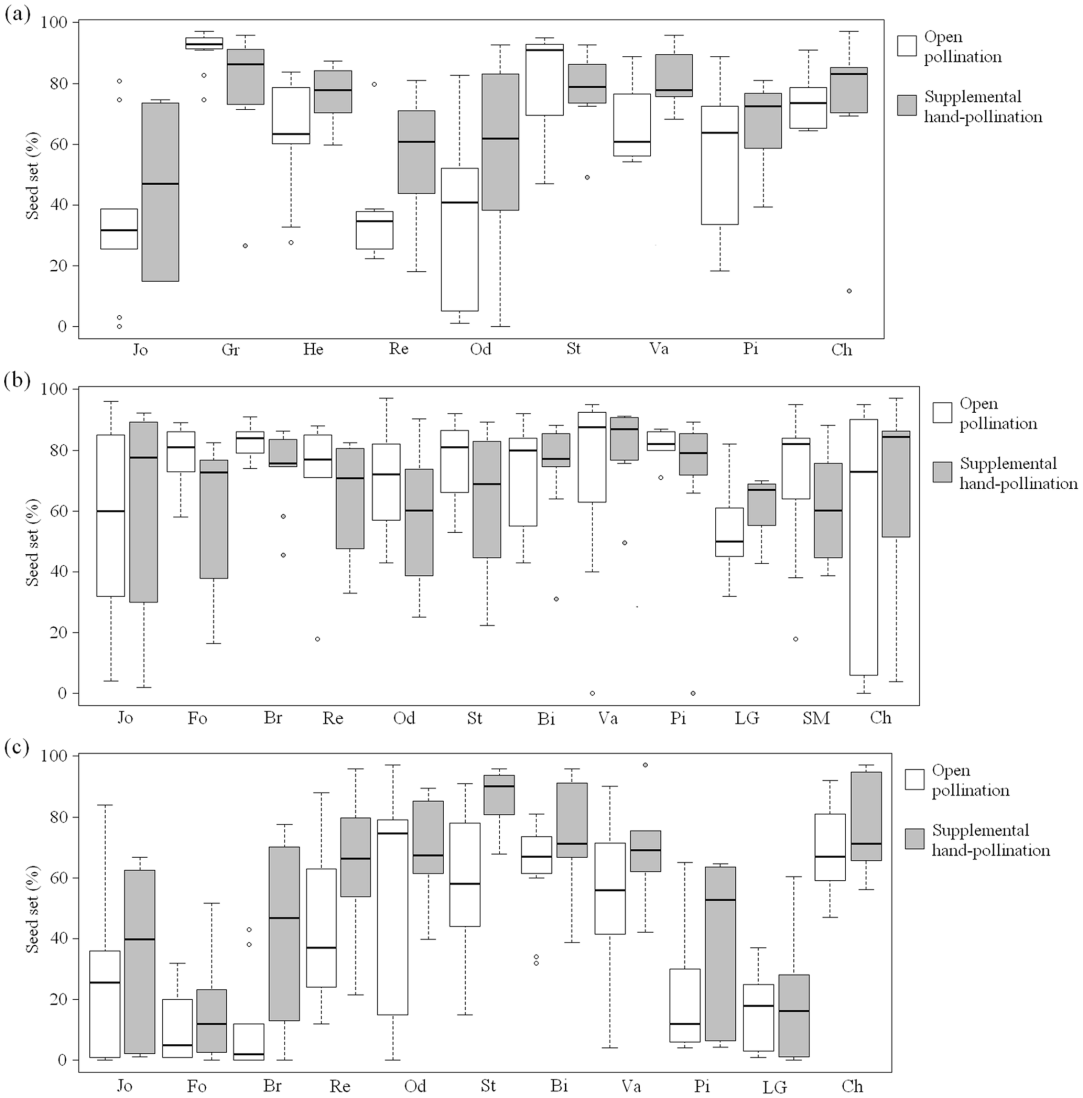
Percentage (%) of viable seeds in fruiting flowers under different pollination treatments. Seed set per fruiting flower following open and supplemental pollination for the 14 Belgian study populations of *Comarum palustre* in (a) 2010, (b) 2011 and (c) 2012. Populations are ordered according to increasing population closure.

On average, fruiting flowers of *Comarum palustre* showed a mean natural seed set of 55.2±1.7%. Seed set differed significantly between pollination treatments (ANOVA: F_1,320_ = 8.49, P<0.005), with a lower seed set resulting from open pollination (55.2±1.7%) than resulting from supplemental pollination (62.0±1.4%; Figure 3).

**Figure 4:**
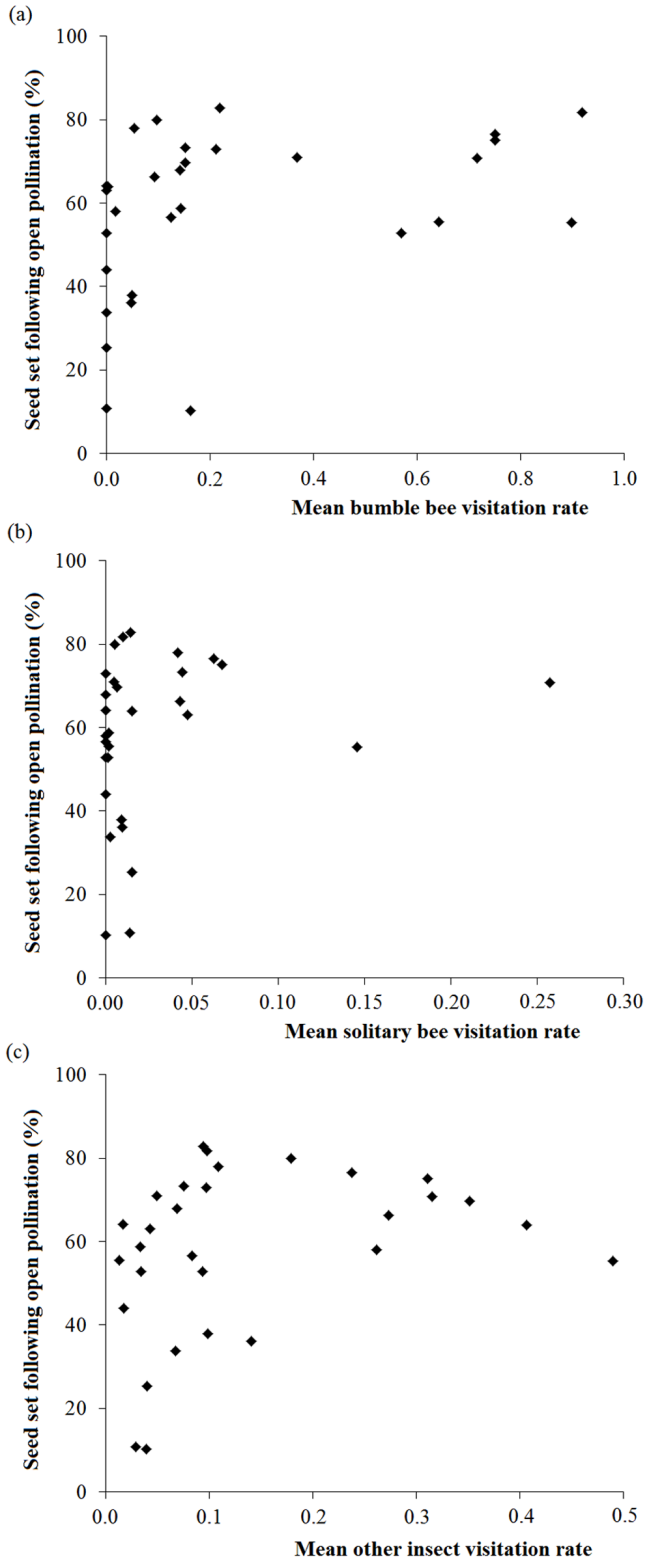
Relationship between insect mean visitation rates and natural seed set. Seed set following open pollination (%) according to the mean visitation rate of (a) bumble bees, (b) solitary bees and (c) other insects. Significant and non-significant results are represented by filled circles and filled triangles, respectively.

Within-population flower density had no effect on seed set resulting from open and supplemental pollinations (Table 3). Also, we did not find any effect of population surface, closure and isolation on seed set (Table 3). Nevertheless, open pollinated flowers from populations with high bumble bee and solitary bee visitation rates showed an increased seed set (Table 3; Figure 4).

**Table 3:** Spatial and pollinator characteristic effects on seed set following pollination treatments.

	Open pollination		Supplemental pollination	
	d.f.	F	d.f.	F
Flower density	1, 279	2.66	1, 279	0.41
Population surface	1, 279	4.00*	1, 279	2.23
Population closure	1, 279	1.66	1, 279	0.98
Population isolation	1, 279	0.43	1, 279	0.22
Bumble bee visitation rate	1, 13	4.74*	1, 13	0.85
Solitary bee visitation rate	1, 13	11.25 ***	1, 13	0.10
Other insect visitation rate	1, 13	1.24	1, 13	3.53

Effect of insect variables (mean number and visitation rate of bumble bees, solitary bees and other insects), within-population flower density and population surface, closure and isolation on seed set resulting from open and supplemental pollination for the 14 *Comarum palustre* populations. (Degrees of freedom (d.f.) and F-values (F) of GLMMs; * P<0.05, ** P<0.01, *** P<0.001).

## Discussion

### Effect of spatial structure on pollinator abundance and visitation rate

We observed that the open, bowl-shaped flowers, easily accessible for pollen and nectar resources, attracted a large variety of insects with 63 different species belonging to eight orders. Our results confirmed that *Comarum palustre* is a generalist species [66], which had already been shown in natural populations in Denmark [47]. Among the observed species, the main visitors of *C. palustre* were bumble bees and solitary bees, supporting previous studies [47], [67].

We found that more bumble bees and other insects, except solitary bees, foraged in high flower density plots within populations. This confirms a previous study on *C. palustre*, where more bumble bees were observed in denser plots than in sparser plots [67]. As bumble bees rely exclusively on pollen and nectar for food and larval provision [56], abundant floral resources at low travelling costs in flower dense plots positively affect their abundance [68]–[70]. It is known that flower density is among the key determinants of pollinator abundance within plant populations [71]–[73].

At the population level, population surface did not affect pollinator abundance nor pollinator visitation rate contrary to Mayer et al. [67], where a positive influence of population surface of *C. palustre* on bumble bee abundance was observed. In this previous study, only four populations were surveyed during one flowering season in 2009, which may underline the importance of studying more populations repeatedly to be able to draw general conclusions.

We further found a positive effect of population closure on bumble bee and solitary bee abundance. Woody elements along wetland edges represent essential foraging zones for emerging bumble bee queens in April and May, since they forage on flowering willows (*Salix* spp.) that are abundant in those areas [74]. The availability of floral resources at emerging time has been reported to positively influence bee nesting densities [74], [75]. Other woody species found at the edges of peat bogs and fens are for instance *Cytisus scoparius* and *Sorbus aucuparia*, that bumble bees included in their food web in late May [68]. Thus, woody edges of wetlands positively influence bee densities [74], [76]. They further provide suitable nesting sites above groundwater level for soil nesting bumble bees [32], [76]–[78] and solitary bees [57], [79], or dead-wood with cavities for wood nesting bees [32]. Another study on the pollination services of *Asclepias lanceolata* reported higher pollinator abundance in the vicinity of woody areas [80]. The other insects (e.g. Coleoptera, Diptera, Lepidoptera and Mecoptera) observed in the study sites showed no response to population closure and are usually considered to be less dependent on nesting opportunities than bumble bees and solitary bees [80], [81]. Although, woody elements at the edge of open areas may buffer microclimatic conditions (temperature, wind and humidity) and might thus offer suitable foraging or resting zones to insects during hot periods [82], [83].

We further found a positive effect of population isolation on the visitation rate of bumble bees and solitary bees. Forests, particularly spruce (*Picea abies*) plantations, represent barriers for bumble bee movements across landscape [38]. Therefore, visits of bumble bees originating from forest edges along the populations of *C. palustre* might be restricted to the isolated plant populations, increasing bumble bee visitation rate. Studies in open landscapes reported bumble bee species foraging up to 2.2 km from their nesting sites, whereas in woody landscapes they usually stay within a radius of 600 m from their nest [30], [84]. The high abundance and visitation rate of solitary bees in isolated populations of *C. palustre* was probably related to their small scale movements, as they usually fly up to 300 m from their nests [35], [57].

### Reproductive success and pollen limitation

Contrary to a former study from Denmark [47], we observed pollen limitation of *C. palustre* since unmanipulated flowers (i.e. left for open pollination) showed a lower seed set than flowers that had received supplemental hand-pollination. As a generalist and self-compatible species, *C. palustre* would be expected to be less vulnerable to pollen limitation [10], [85]. However, its protandrous flowers prevent the species from spontaneous selfing, which makes *C. palustre* dependent on insects for effective pollination [46]. Our results suggested that the visitation rate of solitary bees and bumble bees positively influenced the reproductive success of open pollinated flowers. High visitation rates of pollinators usually increase the chances of pollen deposition on stigmas, mainly through geitono-pollination events (i.e. pollination among flowers within the same genet) in self-compatible species [46], [86], [87]. In particular, bumble bees and solitary bees preferentially forage among near-neighbour flowers, leading to short pollen movements [70]. A decline or the loss of these pollinators might then cause pollen limitation, decreasing the likelihood of successful pollination through compatible pollen deposition, ultimately decreasing plant reproductive success and long-term survival of the plant population [88].

Variation in plant reproductive success might also result from insects foraging both on the target species and other plant species occurring in the surrounding community [89]. Plant community diversity has been reported to influence bee foraging behaviour [76]. Therefore, comparing the attractiveness and the quality of rewards (i.e. pollen and nectar) of plant species within the community might need further consideration in order to disentangle any competition or facilitation effect of the plant community on the reproductive success of *C. palustre*.

## Conclusions

Our findings indicate that within-population flower density, proportion of edges of woody elements and woody areas at the landscape level were determining factors for bee abundance and visitation rate in the studied populations of *C. palustre*. Variations in visitation rate of bumble bees and solitary bees might subsequently influence the reproductive success of the focal plant species. In the current context of wetland fragmentation, it is of major interest to conserve wetlands as foraging zones for insects and further favour multilevel spatial structure mixing woody biotopes with open biotopes, subsequently including nesting sites and food resources for insects. A mosaic of biotopes could thus add to the conservation of plant communities and their interactions with pollinators. Further, a multiple approach should be followed, from within-characteristics of plant populations to spatial structure of landscapes, in order to assess the impact of habitat fragmentation on plant reproductive success. Plant-pollinator interactions and spatial characteristics should thus be considered together.

## Supporting Information

Table S1 **Insect species observed on flowers of** ***Comarum palustre*** **in the 14 study sites from 2010 to 2012.**
(DOC)
