# Supplementary material for: Multilevel Spatial Structure Impacts on the Pollination Services of Comarum palustre (Rosaceae)
Source: PLoS One. 2014 Jun 10;9(6):e99295. doi: 10.1371/journal.pone.0099295 (PMC4051681; doi:10.1371/journal.pone.0099295)
Supplement: Table S1 — Insect species observed on flowers of Comarum palustre in the 14 study sites from 2010 to 2012. (DOC) [file pone.0099295.s001.doc]

Table S1. **Insect species observed on flowers of *Comarum palustre* in the 14 study sites from 2010 to 2012.** The following undetermined species were not included in the table: 1 Coleoptera, 2 Carabidae, 1 Calliphoridae, 1 Muscidae, 1 Syrphidae, 1 Pentatomidae, 1 Eumenidae, 1 Formicidae and 1 Orthoptera species.

| Order | Family | Species |
| --- | --- | --- |
| Coleoptera | Cantharidae | *Cantharis rustica* |
|  |  | *Rhagonycha translucida* |
|  | Cerambycidae | *Leptura rubra* |
|  |  | *Pachytodes cerambyformis* |
|  |  | *Stenurella nigra* |
|  | Oedemeridae | *Oncomera femorata* |
|  |  | *Oedemera nobilis* |
| Diptera | Calliphoridae | *Calliphora vicina* |
|  |  | *Lucilia* sp. 1 |
|  |  | *Lucilia* sp. 2 |
|  | Dolichopodidae | *Dolichopus* sp. |
|  | Muscidae | *Graphomya maculata* |
|  |  | *Phaonia angelicae* |
|  | Stratiomyidae | *Oplodontha viridula* |
|  | Syrphidae | *Anasimyia lineata* |
|  |  | *Eristalis tenax* |
|  |  | *Helophilus pendulus* |
|  |  | *Neoascia geniculata* |
|  |  | *Parelophilus versicolor* |
|  |  | *Rhingia campestris* |
|  |  | *Sphaerophoria menthasti* |
|  |  | *Sphaerophoria scripta* |
|  |  | *Volucella bombylans* |
| Heteroptera | Rhopalidae | *Rhopalus maculatus* |
| Hymenoptera | Andrenidae | *Andrena* sp. 1 |
|  |  | *Andrena* sp. 2 |
|  | Apidae | *Apis mellifera* |
|  |  | *Bombus campestris* |
|  |  | *Bombus hortorum* |
|  |  | *Bombus hypnorum* |
|  |  | *Bombus lapidarius* |
|  |  | *Bombus lucorum* |
|  |  | *Bombus pascuorum* |
|  |  | *Bombus pratorum* |
|  |  | *Bombus terrestris* |
|  |  | *Bombus vestalis* |
|  |  | *Bombus veteranus* |
|  | Colletidae | *Hylaeus biponctatus* |
|  | Halictidae | *Lasioglossum* sp. |
|  | Melittidae | *Macropis fulvipes* |
|  | Tenthredinidae | *Tenthredopsis litterata* |
|  | Vespidae | *Vespula vulgaris* |
| Lepidoptera | Hesperiidae | *Ochlodes venatus* |
|  | Lycaenidae | *Polyommatus icarus* |
|  | Nymphalidae | *Aphantopus hyperantus* |
|  |  | *Boloria eunomia* |
|  |  | *Boloria euphrosyne* |
|  |  | *Brenthis ino* |
|  |  | *Coenonympha pamphilus* |
|  |  | *Maniola jurtina* |
|  |  | *Melitaea diamina* |
|  | Pieridae | *Aporia crataegi* |
| Mecoptera | Panorpidae | *Panorpa Communis* |
